# Skeletal muscle contributions to reduced fitness in cystic fibrosis youth

**DOI:** 10.3389/fped.2023.1211547

**Published:** 2023-06-14

**Authors:** Owen William Tomlinson, Alan Robert Barker, Jonathan Fulford, Paul Wilson, James Shelley, Patrick John Oades, Craig Anthony Williams

**Affiliations:** ^1^ Children’s Health and Exercise Research Centre, Public Health and Sports Sciences, University of Exeter Medical School, University of Exeter, Exeter, United Kingdom; ^2^ Royal Devon University Healthcare NHS Foundation Trust, Exeter, United Kingdom; ^3^ Biomedical and Clinical Science, University of Exeter Medical School, University of Exeter, Exeter, United Kingdom

**Keywords:** exercise capacity, modelling, adolescence, respiratory disease, musculoskeletal

## Abstract

**Background:**

Increased maximal oxygen uptake (V̇O_2max_) is beneficial in children with cystic fibrosis (CF) but remains lower compared to healthy peers. Intrinsic metabolic deficiencies within skeletal muscle (muscle “quality”) and skeletal muscle size (muscle “quantity”) are both proposed as potential causes for the lower V̇O_2max_, although exact mechanisms remain unknown. This study utilises gold-standard methodologies to control for the residual effects of muscle size from V̇O_2max_ to address this “quality” vs. “quantity” debate.

**Methods:**

Fourteen children (7 CF vs. 7 age- and sex-matched controls) were recruited. Parameters of muscle size – muscle cross-sectional area (mCSA) and thigh muscle volume (TMV) were derived from magnetic resonance imaging, and V̇O_2max_ obtained via cardiopulmonary exercise testing. Allometric scaling removed residual effects of muscle size, and independent samples *t*-tests and effect sizes (ES) identified differences between groups in V̇O_2max_, once mCSA and TMV were controlled for.

**Results:**

V̇O_2max_ was shown to be lower in the CF group, relative to controls, with large ES being identified when allometrically scaled to mCSA (ES = 1.76) and TMV (ES = 0.92). Reduced peak work rate was also identified in the CF group when allometrically controlled for mCSA (ES = 1.18) and TMV (ES = 0.45).

**Conclusions:**

A lower V̇O_2max_ was still observed in children with CF after allometrically scaling for muscle size, suggesting reduced muscle “quality” in CF (as muscle “quantity” is fully controlled for). This observation likely reflects intrinsic metabolic defects within CF skeletal muscle.

## 1. Introduction

Higher levels of aerobic fitness [represented by maximal oxygen uptake (V̇O_2max_)] are associated with improved long-term outcomes [e.g., reduced risk of mortality and transplantation (1)] in children with cystic fibrosis (CF). It has further been shown that aerobic fitness is reduced in children with CF compared to healthy peers (2, 3), and whilst many physiological factors are associated with reduced V̇O_2max_ in CF. Precise mechanisms remain unclear in relation to skeletal muscle (4), whose importance has been well established (5).

In particular, debate surrounds whether muscle size (affected by nutritional compromise, attenuated pubertal growth, and catabolism during pulmonary exacerbations) or its intrinsic function [via expression of the CF Transmembrane Conductance Regulator (CFTR) protein within the sarcoplasm (6)] contribute towards skeletal muscle metabolism and reduced V̇O_2max_ in people with CF. This has resulted in a in a muscle “quantity” vs. “quality” debate (7, 8), although these two mechanisms are not guaranteed to be mutually exclusive.

In relation to the “quantity” aspect of this debate, previous studies have expressed V̇O_2max_ relative to body mass and fat-free mass (FFM) using ratio-standard scaling, to account for differences in body size (2, 9, 10). However, this may be inappropriate, as: (a) these variables only provide surrogates for metabolically active muscle during exercise; and (b) the ratio between FFM and leg muscle volume (MV) is not proportional during periods of growth such as puberty (11) and therefore, FFM may be an inferior surrogate of MV during growth. Therefore, accounting for leg MV when assessing V̇O2_peak_ in CF may be more appropriate than body mass and/or FFM as a parameter of body size.

Previous research has tried to account for muscle size in CF when assessing V̇O_2max_, by using ratio-standard scaling of muscle cross sectional area (mCSA), to identify reduced V̇O_2max_ in CF relative to healthy controls (12). However, this is flawed because mCSA poorly represents total leg MV in youth (13), and allometric scaling models have been shown to be more effective in removing residual effects of body size, relative to ratio-standard models (14). Several scaling exponents have been derived previously, all specific to populations being examined (15), indicating the importance of this procedure in eliminating effects of body size from parameters of aerobic fitness, such as V̇O_2max_. Therefore, use of both MV as a scaling factor, and allometry as a scaling method, may better reflect body size when assessing V̇O_2max_ and provide further insight into the quantitative arguments for the “quality vs. quantity” debate.

This study sought to utilise allometric scaling procedures to investigate whether V̇O_2max_ is reduced in CF compared to healthy controls after normalising for thigh MV (TMV) and thigh mCSA. Should V̇O_2max_ remain lower in CF once muscle size is scaled for, this would proffer support for intrinsic muscular defects in this population, as the scaling approach will have accounted for the quantitative effects.

## 2. Methods

### 2.1. Participants & ethics approval

Between 2015 and 2016, seven children with mild-to-moderate CF related lung disease (i.e., forced expiratory volume in one second [FEV_1_] ≥ 70% (16)) were recruited via convenience sampling from routine outpatient clinics and physiotherapy annual review appointments at a local CF centre. Once these participants had completed the study, age- and sex-matched non-CF control children were recruited from a local sports club and state secondary school. An NHS Research Ethics Committee provided ethics approval (14/SW/0061), with written informed consent and assent being obtained from parents/guardians and children respectively prior to participation. Inclusion and exclusion criteria for CF and control groups are provided in Supplementary File 1.

### 2.2. Experimental timeline

Participants underwent two separate visits to the laboratory at the University of Exeter. During an initial familiarisation visit to the laboratory, anthropometric measures, maturity status, lung function, and exercise function were assessed. Participants were also habituated to the magnetic resonance (MR) scanner environment.

### 2.3. Outcome measures

For assessment of maturity status, two approaches were used. Firstly, self-assessment of pubertal status using a validated scale of genital development (17) was undertaken following explanation from a researcher of the same sex, and returned via a sealed envelope. Secondly, via estimated age from peak height velocity (aPHV) using established equations (18), with participants being categorised as “pre-aPHV” or “post-aPHV”.

Lung function, obtained values of FEV_1_ and forced vital capacity (FVC) via hand-held spirometry, with outcomes normalised to global, sex-specific, multi-ethnic, reference values (19).

#### 2.3.1. Cardiopulmonary exercise testing

Participants undertook a cardiopulmonary exercise test (CPET) to volitional exhaustion on an electronically braked cycle ergometer (Lode, Groningen, the Netherlands), using a combined ramp-incremental and supramaximal verification protocol, validated in youth with and without CF (20, 21) to establish a “true” maximal effort (i.e., V̇O_2max_). Breath-by-breath gas exchange data were collected (Cortex Metalyzer, Cranlea, UK), with V̇O_2max_ taken as the highest 10-second average from either the ramp or supramaximal phase, in line with existing validation studies (20, 21). Identification of the gas exchange threshold (GET) was undertaken using the V-slope method (22) and maximal voluntary ventilation (MVV) was estimated by multiplying FEV_1_ by 35 (23). Data for V̇O_2max_ and peak work rate (WR_peak_) was normalised to “percent of predicted” using sex- and modality-specific equations developed in European youth (24).

For children with CF, CPET was undertaken at their physiotherapy annual review, whereby data from this test was obtained retrospectively from medical records to avoid undue burden via multiple tests. For control children, CPET was undertaken in the laboratory at the host institution as previously noted. The same equipment was used in both locations, overseen by the same investigator (OWT), with additional site-specific supervision from staff (PW, JS).

#### 2.3.2. Muscle volume

On the second visit (∼1 week following familiarisation), participants were positioned prone within a 1.5 T superconducting whole-body MR scanner (Gyroscan Intera, Philips, the Netherlands). A T1 weighted image sequence optimised fat/muscle signal contrast, obtaining a stack of axial images from below the knee to above the hip, with TMV and mCSA quantified using methods previously described (13).

#### 2.3.3. Physical activity

Physical activity (PA) was monitored via (non-dominant) wrist-mounted accelerometry for one week (GENEActiv, Activinsights, UK), with time spent being sedentary and in moderate-vigorous activity calculated using age-appropriate cut points (25), and maximal reliability coefficients for minimum daily wear time (26).

#### 2.3.4. Scaling of VO_2max_

Allometric scaling removed residual effects of muscle size from VȮ_max_ and WR_peak_. This process obtains scaling exponents (*b*) and associated 95% confidence intervals (95% CI) from log-linear regressions to use as power functions and to which muscle size is raised (i.e., Y/X^b^), with group included as a covariate within analyses (27). Allometric scaling has been widely used for controlling for body size in relation to interpretation of exercise performance (15), and is a process that has also been used in analysing parameters of aerobic fitness in CF (27).

### 2.4. Statistical analyses

Data are reported as mean ± standard deviation. Pearson's correlation coefficients established relationships between V̇O_2max_ and WR_peak_, and muscle size. Differences between groups were established using independent sample *t*-tests for normally distributed data (determined via Shapiro–Wilk testing), with differences between groups for non-normal data being identified via Mann–Whitney *U* tests. For all tests, *p *< 0.05 indicated statistical significance. Effect sizes (*ES*) using established thresholds from Cohen, 0.2 = (small), 0.5 = (medium), 0.8 = (large) (28), described magnitudes of differences between groups.

## 3. Results

Seven children with CF, and seven age- and sex-matched control children provided data, with a flow chart detailing participation included in Supplementary File 1. Within each group, aPHV data were available for *n *= 6 participants due to non-applicability of equations in females above 16 years of age. For the CF group, *n *= 2 and *n *= 4 were reported as pre- and post-aPHV respectively, and within the control group, *n *= 4 and *n *= 2 were reported as pre- and post-aPHV respectively.

Participants with CF had few comorbidities. All presented as pancreatic insufficient, and one presented with CF related diabetes. None were chronically infected with *Pseudomonas aeruginosa.* Participant characteristics and mean differences between groups for anthropometric, pulmonary, and MR-derived variables are listed in Table 1. Small *ES* were found between groups, with mCSA, TMV, FEV_1_ (%_Pred_) being higher in the CF group.

**Table 1:** Anthropometric, pulmonary, and magnetic resonance-derived muscle-related differences between CF and control groups.

Variable	CF (*n *= 7)	CON (*n *= 7)	*p-*value	Effect size	Minimum (CF, CON)	Maximum (CF, CON)
Sex (Female/Male)	2/5	2/5	–	–	–	
Age (years)	14.8 ± 2.1	14.4 ± 2.2	0.76	0.33	12.1, 11.9	17.5, 17.4
Maturity Stage	4 ± 1^a^	3 ± 1^a^	0.19	**1**.**00**	3, 2	5, 4
Stature (cm)	161 ± 10	162 ± 11	0.88	0.08	141, 146	169, 182
Body mass (kg)	56.7 ± 12.1	52.1 ± 11.0	0.48	0.40	35.5, 39.4	69.6, 62.3
BMI (*z*-score)	0.65 ± 0.51	0.13 ± 0.54	0.09	**0**.**99**	−0.26, −0.91	1.30, 0.69
FEV_1_ (L)	3.32 ± 0.88	3.00 ± 0.73	0.47	0.40	2.04, 2.37	4.83, 4.43
FEV_1_ (%_Pred_)	104.7 ± 11.3	97.2 ± 21.9	0.44	0.43	85.1, 74.6	121.3, 142.6
FVC (L)	3.80 ± 0.99	3.84 ± 0.92	0.95	0.04	2.23, 2.88	5.35, 5.76
FVC (%_Pred_)	103.9 ± 9.5	101.9 ± 11.4	0.74	0.19	90.3, 90.7	116.2, 125.9
FEV_1_/FVC (%)	87.61 ± 4.05	78.50 ± 7.71	**0**.**02**	**1**.**48**	80.3, 66.2	91.5, 91.4
mCSA (cm^2^)^b^	60.8 ± 13.9	56.1 ± 17.6	0.59	0.30	36.4, 40.7	78.7, 86.4
TMV (cm^3^)^b^	2,734 ± 773	2,730 ± 903	0.99	0.00	1,337, 1,701	3,798, 4,276
CFTR Mutations^c^	*Δ*F508/*Δ*F508 (*n* = 3), *Δ*F508/Unknown (*n* = 1), *Δ*F508/E585X (*n* = 1), *Δ*F508/711 + 1G->T (*n* = 1), 18G->T/1–8G->C (*n* = 1)					

Measures are presented as mean ± standard deviation. aPHV, age from peak height velocity; BMI, body mass index; CF, cystic fibrosis; CFTR, cystic fibrosis transmembrane conductance regulator; CON, control; FEV_1_, forced expiratory volume in one second; FVC, forced vital capacity; mCSA, muscle cross-sectional area; TMV, thigh muscle volume. Significant *p*-values (<0.05) in bold. Thresholds for *ES *= 0.2 (small), 0.5 (medium), 0.8 (large), with large *ES* in bold, as per Cohen (28).

^a^ Maturational data only available for 5/7 participants in each group as participants declined to provide information.

^b^ Muscle measures taken from right leg for all participants for consistency in sample.

^c^ CFTR Mutations applicable for CF group only.

Mean differences between groups for exercise data are found in Table 2, and PA data in Table 3. During CPET, 7/14 children obtained their highest V̇O2 in the ramp-incremental phase, 6/14 in the supramaximal phase (within accepted variation (20)), and 1/14 obtained the same value in both exercise bouts.

**Table 2:** Exercise differences between CF and control groups.

Variable	CF (*n *= 7)	CON (*n *= 7)	*p-*value	Effect size	Minumum (CF, CON)	Maximum (CF, CON)
V̇O_2max_ (L.min^−1^)	2.28 ± 0.76	2.58 ± 0.72	0.46	0.41	1.24, 2.02	3.29, 4.04
V̇O_2max_ (mL.kg^−1^.min^−1^)	40.0 ± 8.5	49.7 ± 8.5	0.053	**1**.**14**	27.3, 34.7	51.4, 57.3
V̇O_2max_ (mL.kg^−0.99^.min^−1^)	42.1 ± 9.0	53.5 ± 10.5	0.050	**1**.**17**	28.8, 36.6	54.3, 68.4
V̇O_2max_ (%_Pred_)	87.2 ± 22.9	101.6 ± 12.6	0.17	0.78	57.7, 85.8	128.5, 120.3
WR_peak_ (W)	201 ± 68	208 ± 49	0.83	0.12	101, 164	309, 300
WR_peak_ (W.kg^−1^)	3.49 ± 0.62	4.08 ± 0.50	0.07	**1**.**05**	2.85, 3.37	4.47, 4.82
WR_peak_ (W.kg^−1.25^)	1.26 ± 0.19	1.51 ± 0.19	**0**.**027**	**1**.**32**	1.03, 1.23	1.53, 1.69
WRpeak (%_Pred_)	95.4 ± 22.6	103.3 ± 10.4	0.42	0.45	61.3, 85.1	129.8, 113.8
GET (L.min^−1^)	1.18 ± 0.45	1.25 ± 0.42	0.78	0.16	0.77, 0.95	1.93, 2.09
GET (%V̇O_2max_)	54.4 ± 9.1	49.6 ± 4.4	0.37^d^	0.67	43.0, 41.1	66.9, 53.9
HR_max_ (beats.min^−1^)	182 ± 7^a^	194 ± 7^b^	0.08	**1**.**43**	174, 189	193, 199
V̇_Epeak_ (L.min^−1^)	109.2 ± 46.7	94.5 ± 30.2	0.50	0.37	56.6, 63.5	189.9, 153.0
V̇_E_/MVV (%)	91.9 ± 21.0	95.8 ± 14.3	0.69	0.22	66.2, 73.5	114.9, 114.8
RER_peak_	1.34 ± 0.14	1.14 ± 0.07	**0**.**005**	**1**.**81**	1.17, 1.07	1.53, 1.26
V̇O_2_/WR (mL.W^−1^)	11.4 ± 1.3	12.4 ± 1.7	0.23	0.66	9.1, 10.1	12.5, 14.1

Measures are presented as mean ± standard deviation. CF, cystic fibrosis; CON, control; GET, gas exchange threshold; HR_max_, maximal heart rate; MVV, maximal voluntary ventilation; RER, respiratory exchange ratio; V̇_E_, minute ventilation; V̇O_2max_, maximal oxygen uptake; WR_peak_, peak work rate. Significant *p*-values (<0.05) in bold. Thresholds for *ES *= 0.2 (small), 0.5 (medium), 0.8 (large), with large *ES* in bold, as per Cohen (28).

^a^ HR_max_ data available for 6/7 CF participants due to equipment limitations

^b^ HR_max_ data available for 2/7 CON participants due to equipment limitations.

**Table 3:** Physical activity differences between CF and control groups.

Variable	CF (*n *= 7)	CON (*n *= 7)	*p-*value	Effect size	Minumum (CF, CON)	Maximum (CF, CON)
Sedentary Time (mins)	396 ± 107	441 ± 59	0.35	0.52	270, 354	550, 535
Sedentary Time (%)^a^	54.2 ± 10.1	57.8 ± 7.4	0.47	0.41	45.7, 49.8	72.6, 70.3
Light PA Time (mins)	230 ± 32	208 ± 35	0.44	0.66	144, 159	334, 267
Light PA Time (%)^a^	31.8 ± 7.9	27.1 ± 2.9	0.16	0.79	19.1, 23.1	44.9, 32.1
MVPA Time (mins)	93 ± 33	115 ± 57	0.40	0.47	48, 25	135, 178
MVPA Time (%)^a^	14.0 ± 6.7	15.0 ± 7.5	0.79	0.14	6.2, 3.6	24.4, 25.6
VPA Time (mins)	12 ± 9	13 ± 15	0.48^b^	0.08	3, 0	30, 41
VPA Time (%)^a^	1.9 ± 1.8	1.7 ± 2.0	0.48^b^	0.11	0.3, 0.0	5.7, 5.7

Measures are presented as mean ± standard deviation. CF, cystic fibrosis; CON, control; MVPA, moderate-vigorous physical activity; PA, physical activity; VPA, vigorous physical activity. Significant *p-*values (<0.05) in bold. Thresholds for *ES *= 0.2 (small), 0.5 (medium), 0.8 (large), with large *ES* in bold, as per Cohen (28).

^a^ Physical activity presented as percentage of daily wear time

^b^ *p-*value obtained via Mann–Whitney *U* Test (all other *p-*values from independent samples *t*-test).

V̇O_2max_ was highly correlated with mCSA (CF: *r *= 0.93, *p *< 0.01; control: *r *= 0.92, *p *< 0.01) and TMV in both groups (CF: *r *= 0.84, *p *< 0.05; control: *r *= 0.90, *p *< 0.01). WR_peak_ was also highly correlated with mCSA (CF: *r *= 0.93, *p *< 0.01; control: *r *= 0.96, *p *< 0.01) and TMV (CF: *r *= 0.87, *p *< 0.05; control: *r *= 0.94, *p *< 0.01) in both groups.

In relation to V̇O_2max_, log-linear regression produced scaling exponents for mCSA (*b* = 0.98, 95% CI = 0.66–1.30, *p *< 0.001) and TMV (*b* = 0.78, 95% CI = 0.47–1.10, *p *< 0.001). For WR_peak_, log-linear regression produced scaling exponents for mCSA (*b* = 0.99, 95% CI = 0.67–1.30, *p *< 0.001) and TMV (*b *= 0.81, 95% CI = 0.52–1.10, *p *< 0.001). These exponents successfully removed residual effects of muscle size from V̇O_2peak_ and WR_peak_, evidenced by non-significant Pearson's correlations between scaled V̇O_2peak_ and mCSA (*r *= −0.12, *p *= 0.68) and TMV (*r *= 0.03, *p *= 0.92), and scaled WR_peak_ and mCSA (*r *= −0.13, *p *= 0.65) and TMV (*r *= −0.004, *p *= 0.99).

**Figure 1:**
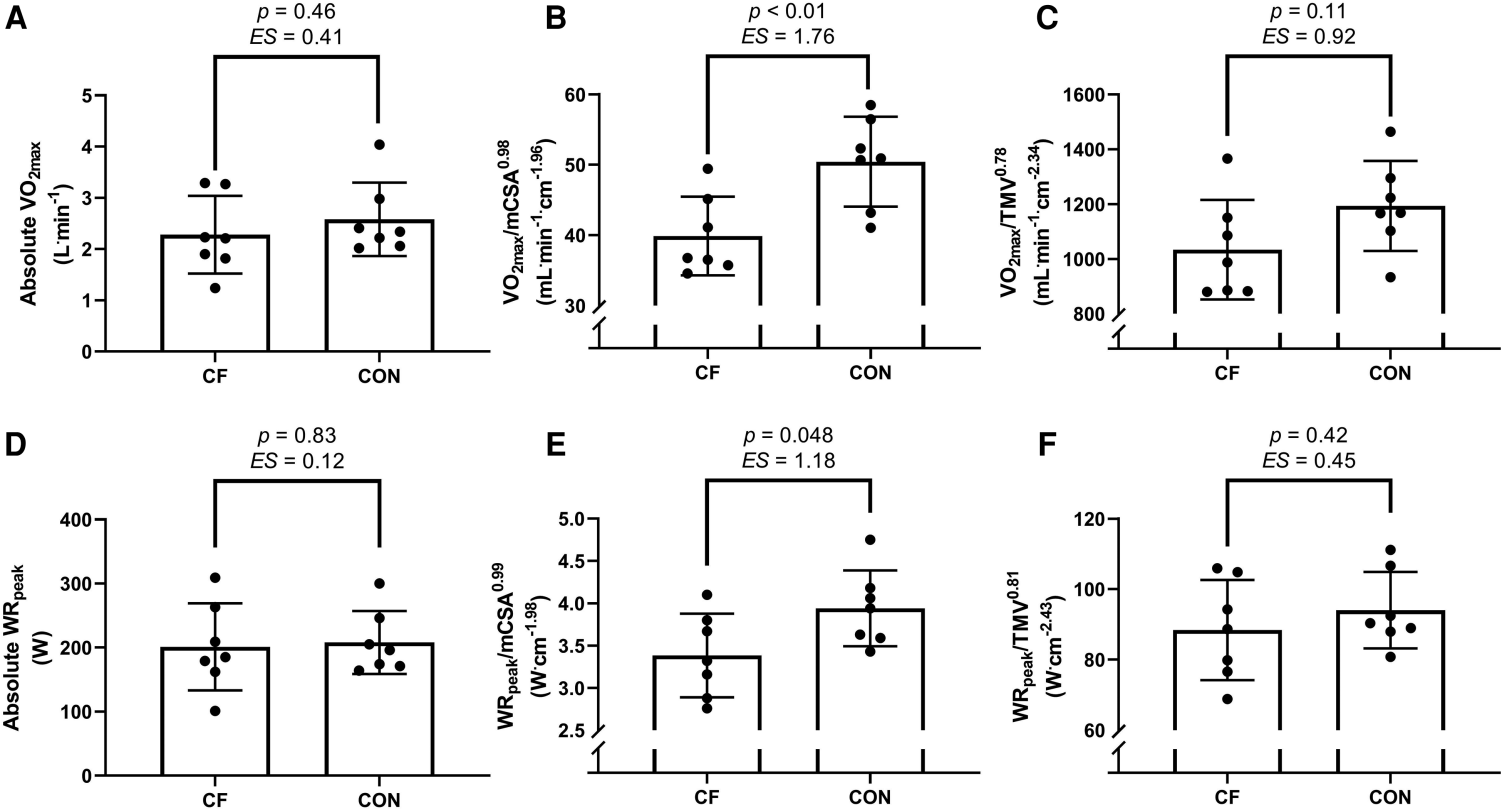
Mean differences between groups for parameters of V̇O_2max_ (**A–C**) and WR_peak_ (**D–F**), presented as an absolute value (**A,D**) and when allometrically scaled to muscle cross sectional area (mCSA) of the right mid-thigh (**B,E**) and to thigh muscle volume (TMV) of the right leg (**C,F**). CF, cystic fibrosis; CON, control; *ES*, effect size; V̇O_2max_, maximal oxygen uptake, WR_peak_, peak work rate. Data presented as mean ± standard deviation. Thresholds for *ES* = 0.2 (small), 0.5 (medium), 0.8 (large) as per Cohen (13).

Therefore, once these exponents were applied, a large *ES* was found between groups for both mCSA and TMV scaled V̇O_2max_, being higher in the control group (Figure 1). When used to scale WR_peak_, a large *ES* was found between groups for mCSA but not TMV, but both values being higher in the control group (Figure 1).

## 4. Discussion

The main result of this study has shown that when parameters of muscle size are allometrically scaled for, a large difference (as shown by *ES*) in V̇O_2max_ is found, whereby this is higher in the control group relative to CF. Thus, after robustly accounting for muscle “quantity”, a difference remains between groups and thus supports the case for muscle “quality” accounting for the reduced V̇O_2max_ (and WR) in CF.

Previous work indicates a strong correlation (*r *= 0.89) between mCSA and V̇O_2max_ in CF (12), agreeing with the coefficient found within the present work (*r *= 0.93). Moreover, the present investigation characterised the association between V̇O_2max_ and TMV for the first time in CF, finding an equally large coefficient between variables (*r *= 0.84); a result that reflects prior findings in healthy children (29). These coefficients show a clear relationship between parameters, and therefore use of muscle size to scale V̇O_2max_ is warranted.

In identifying reduced V̇O_2max_ relative to age- and sex-matched peers, these findings corroborate previous research that identifies similar reductions in V̇O_2max_ in children with CF when body mass and FFM (30) are accounted for. However, as previously noted, body mass and FFM are poor surrogates for metabolically active muscle (11) and therefore quantification of muscle size should be considered instead. Using muscle size, the present work supports previous findings that utilised mCSA (with a very similar scaling exponent of *b *= 1.03 to the present *b *= 0.98) to scale V̇O_2max_ and find increased fitness in control children relative to those with CF (12). Moreover, when also scaling for TMV, the present work found a broadly similar scaling exponent (*b *= 0.78) to that previously found in healthy children (29) (*b *= 0.55; the present 95% CI encompasses this value). Within the present study, both groups presented with remarkably similar mean values, and thus when the scaling exponent is applied, collective findings of previous (12) and present work indicate “qualitative” defects in CF skeletal muscle once “quantitative” differences are controlled.

These novel, methodologically robust data, supports studies conducted *in vitro* that show that CFTR affects mitochondrial function, resting adenosine triphosphate levels and Ca^2+^ regulation (6, 31). Moreover, *in vivo* research corroborates these findings as CFTR expression within skeletal muscle is associated with upregulation of genes responsible for muscle atrophy (32) and prolonged phosphocreatine recovery following exercise (33). In addition, prior work undertaken in adolescents with CF indicates that exercise intolerance may be intensity-dependent, whereby moderate-intensity exercise does not differ between groups, but it is in the high-intensity domain where differences in oxidative metabolism emerge (30). This would be supported by the current work, as exercise was performed at maximal levels during CPET.

The data presented within the current study indicates a “qualitative” defect within skeletal muscle, adding evidence to the “quality” side of the “quality vs*.* quantity” debate (7, 8). However, contrasting studies show that muscle power is not different between people with CF and healthy controls when muscle size is accounted for (34, 35). However, muscular power, in these studies, has been obtained via Wingate testing (34) and via plantar flexion testing (35), and not via CPET. However, V̇O_2peak_ remains impaired in these studies, thus evidencing that skeletal muscle “quality” likely impairs oxidative metabolism, if not force generation. Moreover, these prior studies (34, 35) include heterogenous patient groups with greater levels of pulmonary impairment and comorbidities, contrasting the present work whereby the relatively good health status of the CF group ensures that no additional disease associated complications (poor lung function, bacterial infection, sarcopenia, hypoxemia) confound results.

Whilst this study has a limited sample size (typical of research in clinical populations and those using costly MR techniques), several strengths remain. Notably, age- and sex-matching of participants ensured disease status remained the discerning characteristic between groups. Whilst matching via calendar age alone is flawed, particularly in youth, assessments of maturity status ensured that groups were broadly matched in terms of biological and somatic maturation as well. Moreover, participants with CF had preserved pulmonary function, as well as similar exercise function (i.e., V_E_/MVV, VO_2_/WR), physical activity status and muscle size parameters, and yet V̇O_2max_ was still reduced relative to controls. This observation further indicates that predominantly skeletal muscle defects, and not necessarily pulmonary or cardiovascular factors, likely affect V̇O_2max_ in CF.

High quality physiological techniques were used in this study, including gold-standard CPET and associated supramaximal verification bouts (21). The choice of the latter being vindicated by the observation that 43% of children achieved their highest V̇O_2_ during the supramaximal verification bout, thus enhancing the evidence base for why this additional verification is needed in paediatric studies. In addition to gold-standard CPET, MR imaging derived measures of mCSA and MV, and robust allometric scaling aided in the removal of residual effects of muscle size. All these testing and analytical procedures combined to create a robust design and analyses.

With regards to limitations, it is acknowledged that an element of selection bias may be present, as children were selected from a singular paediatric clinic in the UK. Therefore, it is possible that children who enjoy exercise were more likely to volunteer for an exercise-based study and thus the lack of individuals with more severe disease in this study does not necessarily reflect the wider, heterogenous, CF community. This is reflected by participants lacking chronic infection, as well as data in Tables 1, 3, whereby mean FEV_1_ > 100%_Pred_, and mean MVPA equated to >90 min per day, respectively. However, even with selection bias, and inclusion of nominally healthy and active children with CF, we still observed a large effect size between groups, indicating an inherent CF-related cause to exercise intolerance, which may become even more pronounced if children with less stable CF were included. We therefore call for replication of the current work to replicate these initial findings.

Finally, we acknowledge this work was conducted prior to the widespread introduction of CFTR modulators that may affect exercise function (4). However, as not all patients are eligible for modulator therapy either through ineligible genotype, clinical access arrangements, global cost and differences in healthcare systems or adverse reactions (36). Additionally, as there is no association between genotype and fitness (37) (which may be impacted by modulators if an association existed), the current findings retain applicability to a wide number of people with CF and to the continued exercise management of the disease. The subsequent impact of CFTR modulator therapy upon skeletal muscle function should be examined in this group to provide additional mechanistic insight into this “quality vs*.* quantity” debate.

## 5. Conclusion

In summary, through the use of gold-standard CPET and MR procedures, this study has found a reduced V̇O_2max_ in children with CF once muscle size is fully accounted for, thus furthering evidence for the proposed intrinsic muscular defect in this population (7). Whilst this data does not wholly confirm a “qualitative” defect and that muscle “quality” and “quantity” may not be mutually exclusive, we recommend rehabilitation programmes and exercise training regimens should consider improvements in both muscle “quality” and “quantity” for people with CF.

## Data Availability

The raw data supporting the conclusions of this article will be made available by the authors, without undue reservation.
