# Supplementary material for: Skeletal muscle contributions to reduced fitness in cystic fibrosis youth
Source: Front Pediatr. 2023 Jun 14;11:1211547. doi: 10.3389/fped.2023.1211547 (PMC10300555; doi:10.3389/fped.2023.1211547)
Supplement: Supplementary file 1 [file Datasheet1.pdf]

SUPPLEMENTAL FILE 1. Participant Criteria and Flow Chart

Table 1. Inclusion and exclusion criteria for people with cystic fibrosis and healthy controls

	Cystic Fibrosis	Control
Inclusion Criteria	• Male and females aged 10-18 years diagnosed with CF<br>• CF diagnosis based on clinical features, supported by an abnormal sweat test (sweat chloride > 60 mmol·L^{-1} > 100 mg sweat), where possible, diagnostic genotyping would also be desired<br>• Child is regularly participating in physical activity<br>• Child presents with no contraindications to performing exhaustive exercise within an MR scanner<br>• Child can understand and cooperate with the study protocol<br>• Lung function considered stable and within 10 % of their best in the preceding 6 months<br>• No increase in symptoms or weight loss in the preceding 2 weeks	• Healthy males and females aged 10-18 years who are age- and gender-matched to the chest disease patients<br>• No diagnosis of chest disease or asthma<br>• Child is regularly participating in physical activity<br>• Child presents with no contraindications to performing exhaustive exercise within an MR scanner<br>• Child can understand and cooperate with the study protocol
Exclusion Criteria	• Any non-pulmonary conditions that may impair exercise ability, such as musculoskeletal disorders (active arthritis, joint or muscle disease) and cardiovascular disease (congenital heart disease or cardiomyopathy).<br>• Unstable co-morbid asthma (daily PF variability of >20 %)<br>• Child presents with co-morbidities to performing exhaustive exercise within an MR scanner<br>• Unable to understand or cooperate with the study protocol due to learning difficulties or otherwise<br>• <10 years of age<br>• >18 years of age<br>• Onset of acute infection<br>• Child is not happy being within the MR scanner environment	• Any pulmonary conditions<br>• Any non-pulmonary conditions that may impair exercise ability, such as musculoskeletal disorders (active arthritis, joint or muscle disease) and cardiovascular disease (congenital heart disease or cardiomyopathy).<br>• Child presents with co-morbidities to performing exhaustive exercise within an MR scanner<br>• Unable to understand or cooperate with the study protocol due to learning difficulties or otherwise<br>• Not an age- or gender-match for the chest diseased participants<br>• <10 years of age<br>• >18 years of age<br>• Child is not happy being within the MR scanner environment

CF, Cystic Fibrosis; MR, magnetic resonance; PF, pulmonary function

Figure 1. Participation flow chart.

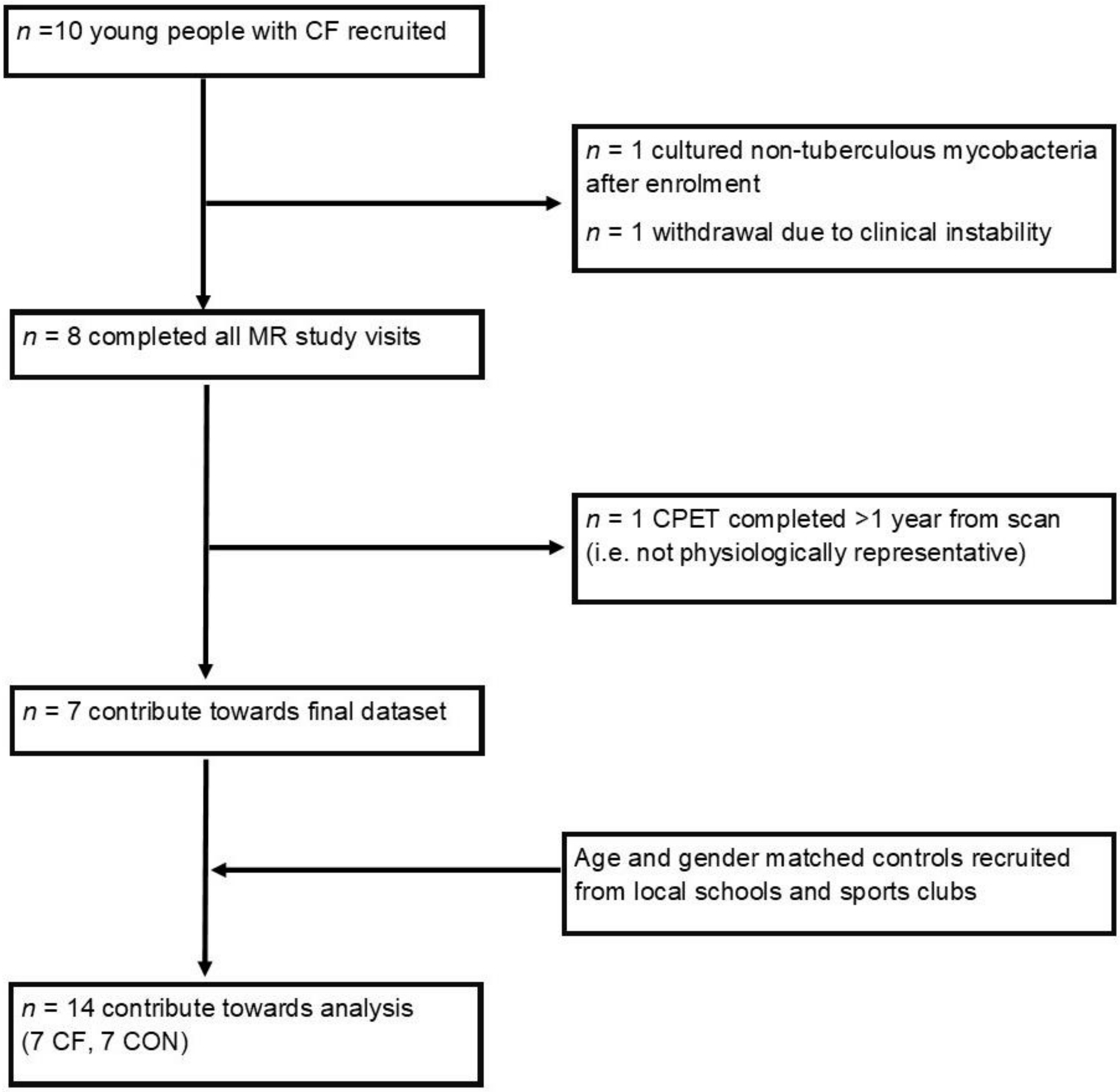

CF, cystic fibrosis; CON, control participant; CPET, cardiopulmonary exercise test; MR, magnetic resonance
